# The Role of Acetate Kinase in the Human Parasite *Entamoeba histolytica*

**DOI:** 10.3390/parasitologia2020014

**Published:** 2022-06-16

**Authors:** Thanh Dang, Matthew Angel, Jin Cho, Diana Nguyen, Cheryl Ingram-Smith

**Affiliations:** 1 Department of Genetics and Biochemistry, Clemson University, Clemson, SC 29634, USA; 2 Eukaryotic Pathogens Innovation Center, Clemson University, Clemson, SC 29634, USA

**Keywords:** *Entamoeba histolytica*, acetate kinase, glycolysis

## Abstract

The human parasite *Entamoeba histolytica*, which causes approximately 100 million cases of amoebic dysentery each year, relies on glycolysis as the major source of ATP production from glucose as it lacks a citric acid cycle and oxidative phosphorylation. Ethanol and acetate, the two major glycolytic end products for *E. histolytica*, are produced at a ratio of 2:1 under anaerobic conditions, creating an imbalance between NADH production and utilization. In this study we investigated the role of acetate kinase (ACK) in acetate production during glycolysis in *E. histolytica* metabolism. Analysis of intracellular and extracellular metabolites demonstrated that acetate levels were unaffected in an *ACK* RNAi cell line, but acetyl-CoA levels and the NAD^+^/NADH ratio were significantly elevated. Moreover, we demonstrated that glyceraldehyde 3-phosphate dehydrogenase catalyzes the ACK-dependent conversion of acetaldehyde to acetyl phosphate in *E. histolytica*. We propose that ACK is not a major contributor to acetate production, but instead provides a mechanism for maintaining the NAD^+^/NADH balance during ethanol production in the extended glycolytic pathway.

## 1. Introduction

*Entamoeba histolytica* is an amoebic parasite that causes diarrheal illness in an estimated 100 million people worldwide [1] as well as amoebic liver abscess that results in 50,000–100,000 deaths annually [2]. Infection proceeds through an oral–fecal route, resulting in *E. histolytica* colonization within the large intestine [3,4]. This amitochondriate parasite lacks many essential biosynthetic pathways including the citric acid cycle and oxidative phosphorylation, and glycolysis is the primary pathway for ATP generation during growth on glucose [5,6]. Unlike the standard glycolytic pathway, *E. histolytica* glycolysis is pyrophosphate (PP_i_)-dependent. Instead of ATP–dependent phosphofructokinase and pyruvate kinase, *E. histolytica* possesses PP_i_–dependent phosphofructokinase and pyruvate phosphate dikinase [7–10]. Pyrophosphate, therefore, plays an important role in energy conservation.

**Figure 1.**
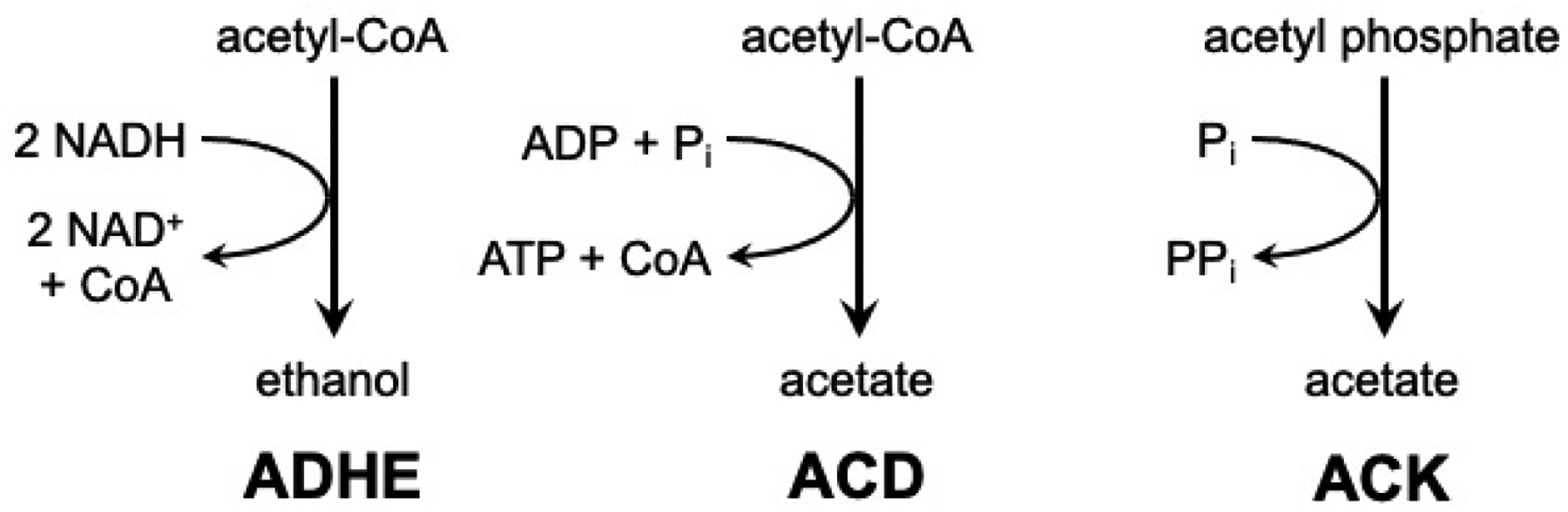
Ethanol and acetate production in *E. histolytica*. ADHE (**left**) catalyzes production of ethanol from acetyl-CoA. ACD (**middle**) and ACK (**right**) produce acetate from acetyl-CoA and acetyl phosphate, respectively.

Ultimately, *E. histolytica* produces ethanol and acetate as the major end products during growth on glucose [11,12]. Ethanol is produced by the bifunctional alcohol/aldehyde dehydrogenase ADHE in a two-step pathway (Figure 1) [13–15]. *E. histolytica* has two potential acetate-producing enzymes, acetate kinase (ACK) [16,17] and ADP-forming acetyl-CoA synthetase (ACD) (Figure 1) [12,18]. ACK (EC 2.3.1.8) is a phosphotransferase that interconverts acetyl phosphate and acetate. It is widespread in bacteria where it primarily functions in a pathway with phosphotransacetylase (PTA; EC 2.3.1.8) to activate acetate to acetyl-CoA or produce acetate and ATP from acetyl-CoA [19]. In the archaeal genus *Methanosarcina* ACK forms a pathway with PTA for the activation of acetate as a substrate for methane production [20,21]. ACK, previously thought to be absent in eukaryotes, has now been identified in euascomycete and basidiomycete fungi in which it forms with xylulose 5-phosphate/fructose 6-phosphate phosphoketolase (XFP; EC 4.1.2.22) for the production of acetate [22]. ACK partners with PTA in the green alga *Chlamydomonas reinhardtii* [23] and the oomycete *Phytophthora* [22].

ACK has also been identified in *Entamoeba* and characterization of the recombinant *E. histolytica* ACK (EhACK) revealed it to be phosphate/pyrophosphate (P_i_/PP_i_)-dependent, a stark contrast to all other characterized ACKs which are ATP/ADP-dependent [16,17]. EhACK operates primarily in the acetate/PP_i_-producing direction in vitro [16,17]. Consistent with this, activity was detected only in the acetate/PP_i_-producing direction in cell extract [16,17], supporting this as the physiological direction of EhACK. However, the role of ACK in *E. histolytica* still remains undefined and a partner enzyme that produces the acetyl phosphate substrate has not yet been identified. Harting and Velick [24,25] demonstrated that rabbit muscle glyceraldehyde 3-phosphate dehydrogenase (GAPDH) can produce acetyl phosphate from acetaldehyde, raising this as a possible new partner for ACK.

Here, we investigated the physiological role of ACK in *E. histolytica* and evaluated whether EhACK is the primary enzyme responsible for acetate production during growth on glucose. Growth of an *ACK* RNAi cell line was unaffected versus the wild-type and a control RNAi cell line and extracellular acetate and ethanol levels were comparable between the three cell lines. Intracellular ATP levels were also unaffected, but the *ACK* RNAi cell line had higher intracellular acetyl-CoA and an increased NAD^+^/NADH ratio. We demonstrated that recombinant *E. histolytica* GAPDH (EhGAPDH) can use acetaldehyde as a substrate, but only in the presence of ACK. Based on this evidence, we propose that EhACK functions to maintain the proper NAD^+^/NADH balance for ethanol production during glycolysis and is not a major contributor to acetate production.

## 2. Results

### 2.1. Trigger-Mediated ACK Gene Silencing

**Figure 2.**
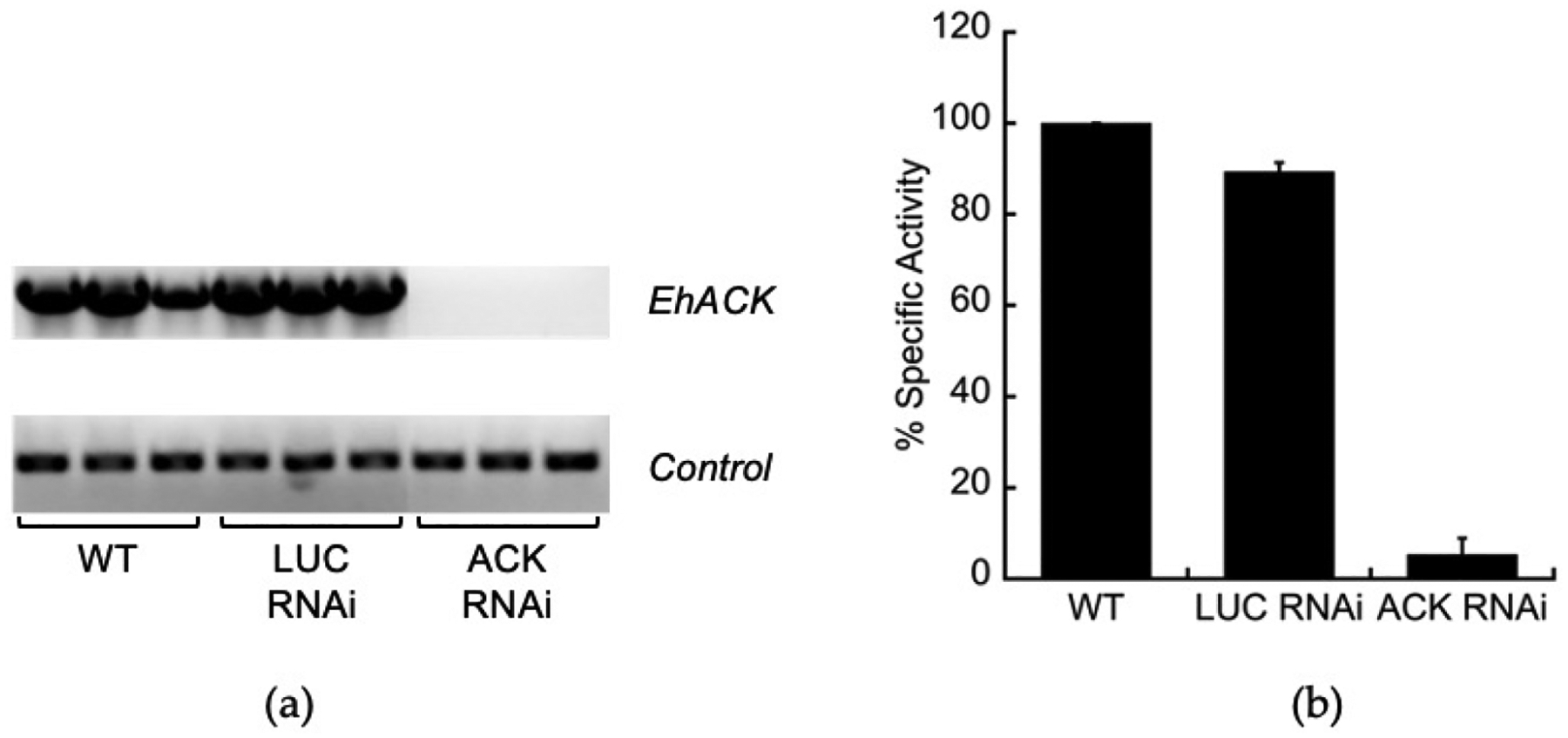
ACK expression and activity in the *EhACK* RNAi cell line. (**a**) Expression of *EhACK* in the wild-type (WT), control LUC RNAi, and *EhACK* RNAi cell lines was examined by RT-PCR (top). Expression of a constitutive control gene encoding a small ribosomal subunit was used as a loading control (bottom). Each RT-PCR reaction was performed in triplicate. (**b**) ACK activity in the acetate-forming direction was measured using the reverse hydroxamate assay. Activities are the mean ± standard deviation of three replicates. All specific activities are normalized to that observed for extracts from the wild-type, represented as 100%.

To investigate the role of ACK in *E. histolytica*, we created an *ACK* RNAi cell line to examine the effect of *ACK* gene silencing on growth and metabolism. The *EhACK* gene was silenced using the trigger-mediated small antisense gene silencing method developed by Morf et al. [26]. The pKT3M vector used for this has a resident luciferase gene (*LUC*) and is used as a control. The *EhACK* coding sequence was cloned into the pKT3M vector to replace the resident luciferase gene (*LUC*) and transfected into wild-type *E. histolytica*. A *LUC* RNAi cell line was also constructed to be used as a control cell line that would be under the same G418 selection as the *ACK* RNAi cell line. RT-PCR was performed to examine ACK transcript levels in wild-type, the control *LUC* RNAi cell line, and the *ACK* RNAi cell line. *ACK* mRNA levels were comparable in the wild-type and the *LUC* RNAi cell line but undetectable in the *ACK* RNAi cell line (Figure 2a). Enzymatic assays demonstrated that ACK enzymatic activity was reduced 18-fold in the *ACK* RNAi cell line but was unaffected in the control *LUC* RNAi cell line (Figure 2b). Taken together, these results confirmed successful *ACK* silencing in the *ACK* RNAi cell line.

### 2.2. ACK Is Dispensable for Growth on Glucose

**Figure 3.**
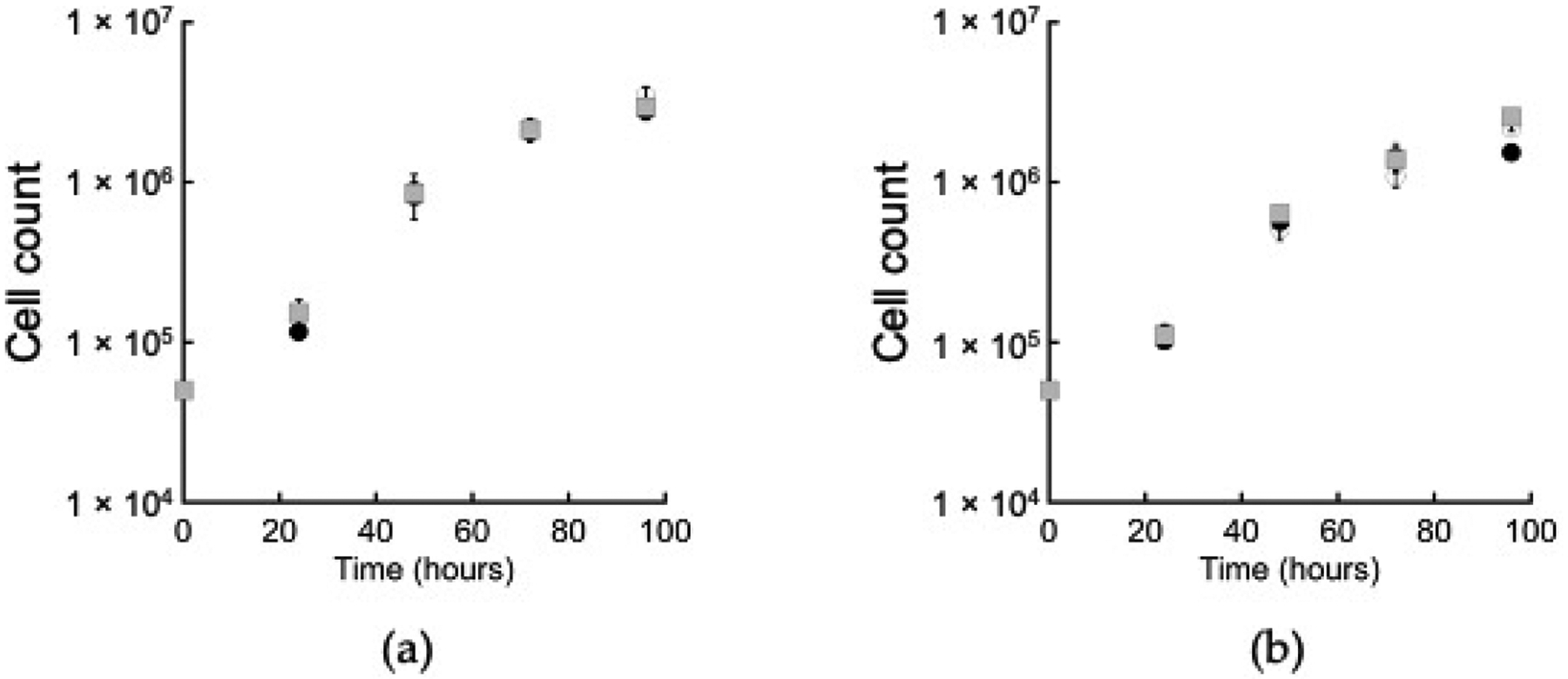
Growth of the *ACK* RNAi cell line in (**a**) TYI glucose medium, and (**b**) TYI low glucose medium. Open circles (○), wild-type; closed circles (●), *LUC* RNAi cell line; gray squares (■), *EhACK* RNAi cell line. Cell counts are the mean ± standard deviation of three biological replicates.

One potential role for ACK is in acetate production in an extended glycolytic pathway. Acetate and ethanol are the main end products during *E. histolytica* growth on glucose [11,12] and we hypothesized that if ACK plays a primary role in acetate production, then an *ACK* RNAi cell line would have impaired growth on glucose. We measured growth in standard TYI-S-33 medium, which contains 50 mM added glucose (designated here as TYI glucose), and in TYI-S-33 medium in which the added glucose has been lowered to 10 mM (designated here as TYI low glucose). Growth of the wild-type, *LUC* RNAi, and *ACK* RNAi cell lines was similar at both glucose levels (Figure 3).

### 2.3. Intracellular but Not Extracellular Metabolite Levels Are Altered in the ACK RNAi Cell Line

**Figure 4.**
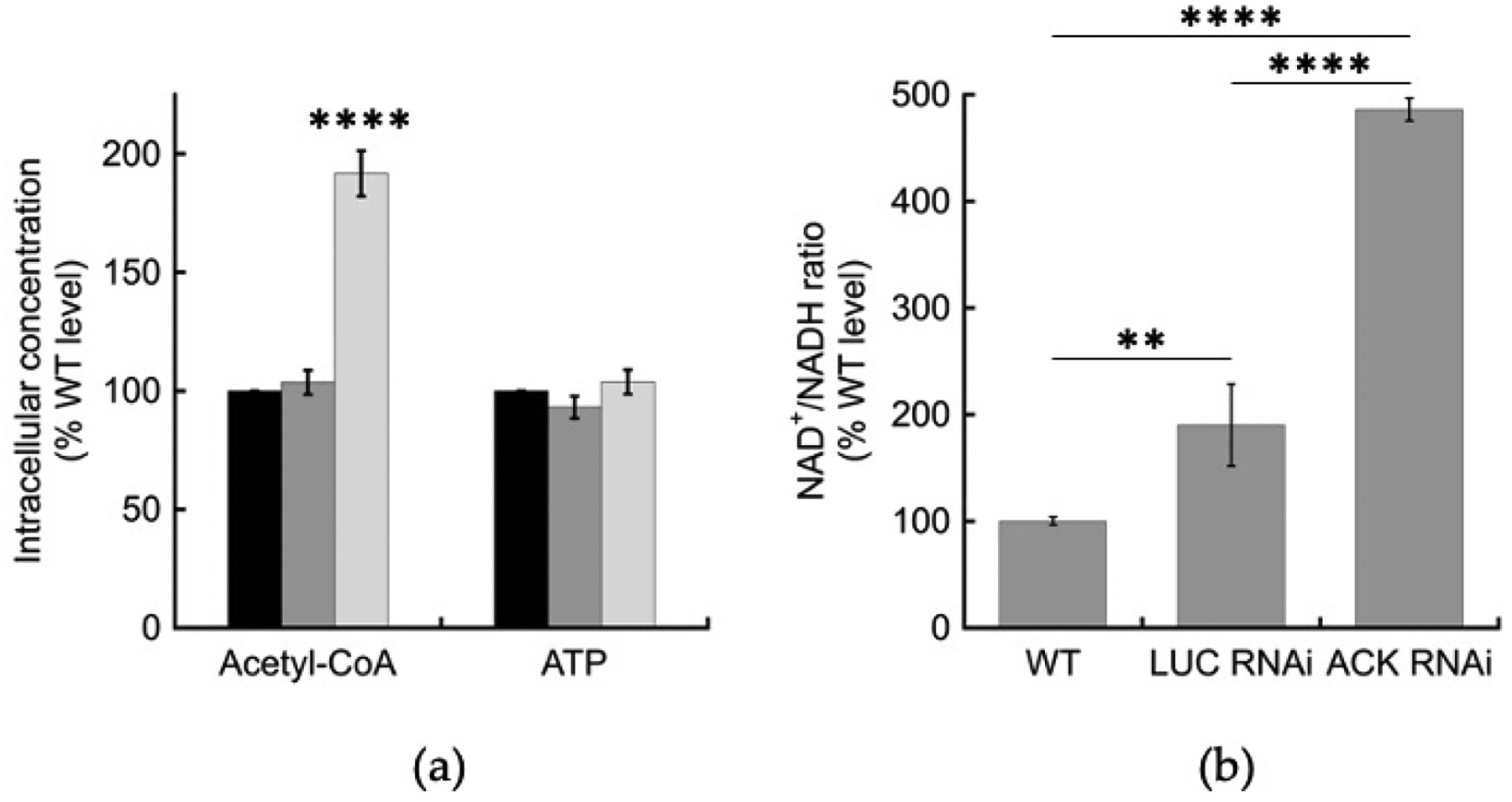
Intracellular metabolite levels in *ACK* RNAi cells. Metabolites were extracted using methanol extraction from log-phase trophozoites and concentrations were measured using LC-MS/MS. (**a**) Concentrations of acetyl-CoA and ATP. Wild-type (■), *LUC* RNAi (■), *EhACK* RNAi cells (■). (**b**) Ratio of NAD^+^/NADH, normalized to wild-type ratio. Measurements are the mean ± standard deviation of three to five replicates. ** *p*-value ≤ 0.01; **** *p*-value ≤ 0.0001.

To evaluate EhACK’s role in glycolysis, we determined intracellular acetyl-CoA and ATP levels in cells grown 48 h in TYI glucose medium as measures of glycolytic activity and intracellular energy levels. The *ACK* RNAi cell line showed an accumulation of acetyl-CoA, with a level ~172% that of the wild-type and the *LUC* RNAi cell line, but the intracellular ATP level was unaffected (Figure 4a). The *ACK* RNAi cell line also experienced ~2.5–fold increased NAD^+^/NADH ratio versus that observed for the *LUC* RNAi cell line and ~4.9-fold higher than for the wild-type cell line (Figure 4b). Intracellular NADH was reduced in the both the *LUC* RNAi and *ACK* RNAi cell lines to 56.3 ± 25.0% and 21.9 ± 0.3% that of the wild-type, respectively. One possible explanation for why the *LUC* RNAi cell line had a higher NAD^+^/NADH ratio than observed in the wild-type is that it is under G418 selection. However, the *ACK* RNAi cell line, also under G418 selection, still exhibited a significantly higher NAD^+^/NADH ratio than the *LUC* cell line.

**Figure 5.**
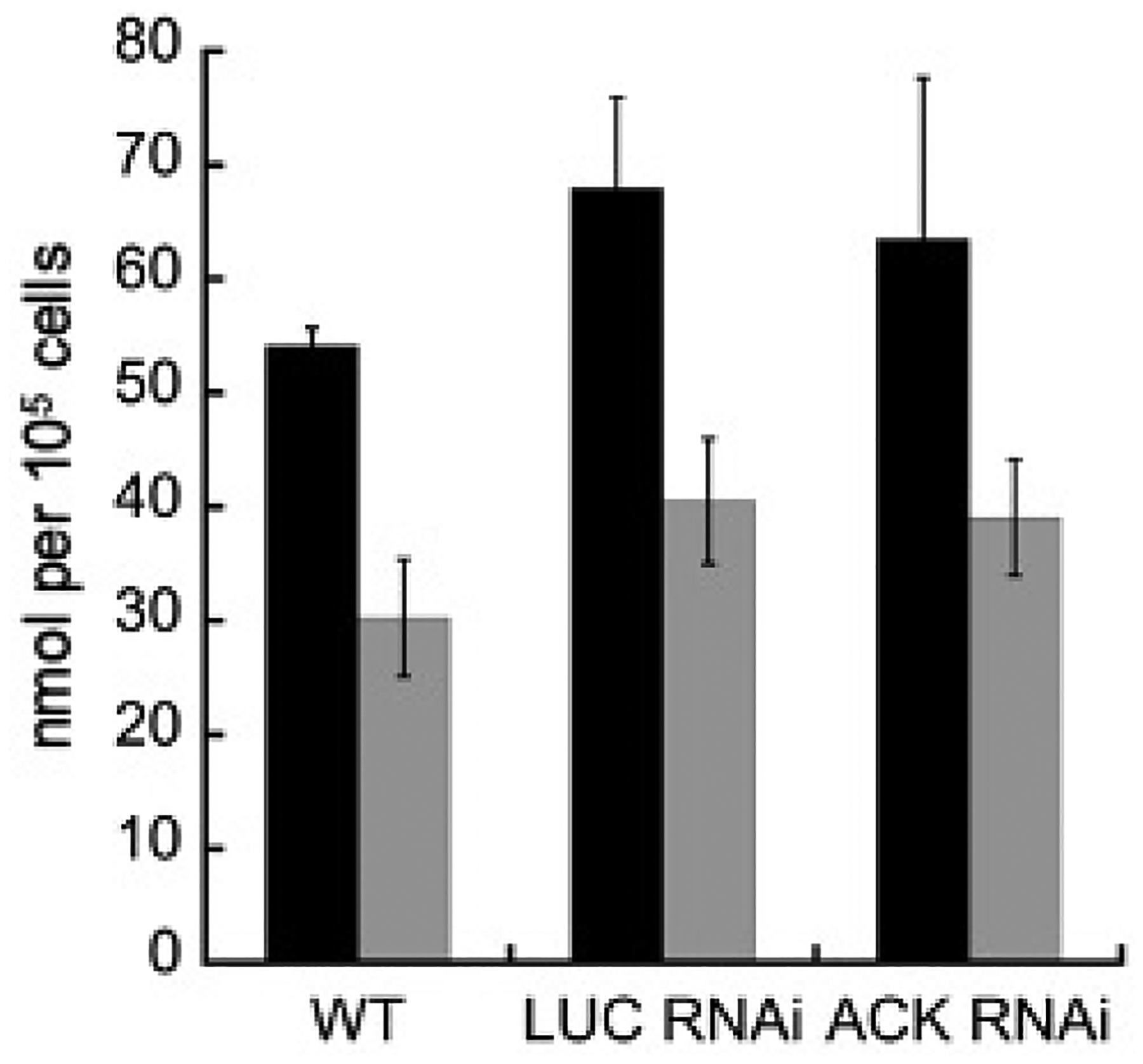
Extracellular ethanol and acetate levels in spent TYI glucose medium. Ethanol (■) and acetate (■) were measured in spent medium from cultures of wild-type, *LUC* RNAi, and *ACK* RNAi cell lines grown in TYI glucose medium for 48 h. The results shown represent the mean ± standard deviation for four to five biological replicates for each cell line. Unpaired *t*-test revealed no statistical difference between cell lines for either ethanol or acetate.

The ratio of extracellular ethanol to acetate produced by *E. histolytica* during growth on glucose varies depending on oxygen level [11,12]. To determine if ACK plays a significant role in the production of acetate and impacts the ethanol:acetate ratio, we measured extracellular acetate and ethanol in spent medium from the wild-type, *LUC* RNAi, and *ACK* RNAi cell lines grown for 48 h in TYI glucose medium (Figure 4). Production of acetate and ethanol was similar in the *ACK* RNAi cell line versus the wild-type cell line or the *LUC* RNAi control cell line (Figure 5). The calculated ratios of ethanol to acetate were found to be 1.8:1, 1.7:1, and 1.6:1 for the wild-type, *LUC* RNAi, and *ACK* RNA cell lines, respectively.

### 2.4. ACK Does Not Play a Role in Utilization of Short Chain Fatty Acids

**Figure 6.**
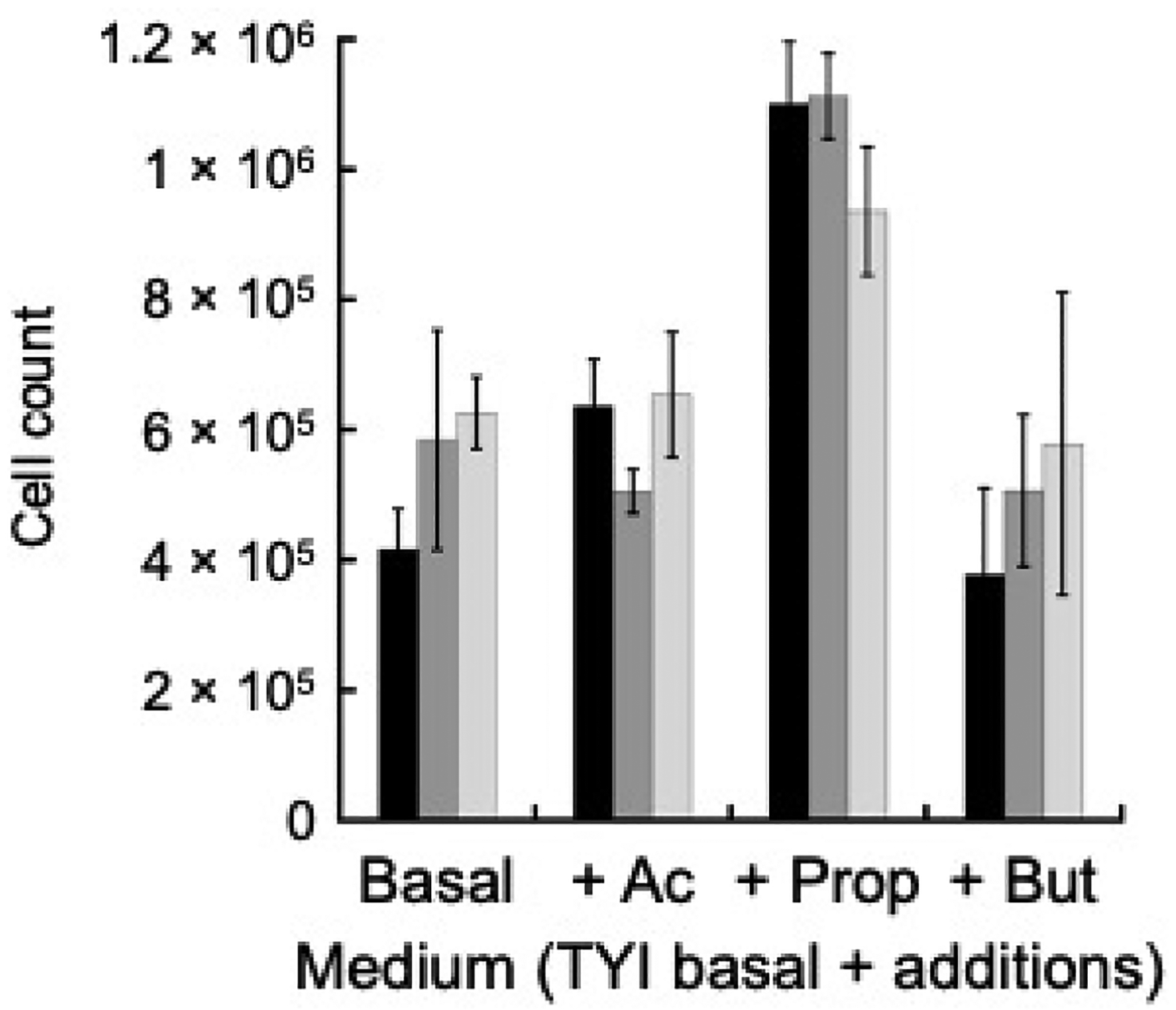
Growth of *EhACK* RNAi cells in basal medium supplemented with short chain fatty acids (SCFAs). Cells were grown for 72 h in TYI basal medium or TYI basal medium supplemented with acetate (Ac), propionate (Prop), or butyrate (But). Wild-type (■), *LUC* RNAi (■), *EhACK* RNAi cells (■). Cell counts are the mean ± standard deviation of three biological replicates.

*E. histolytica* colonizes the human colon, where glucose is scarce as most dietary glucose is absorbed in the small intestine. Short chain fatty acids (SCFAs) are abundant though with a total concentration of 110–120 mM mainly consisting of acetate, propionate, and butyrate at a relative molar mass ratio of 57:22:21, respectively [27]. Although kinetic analysis showed EhACK strongly favors acetate production [16,28], we examined whether it plays a role in growth on SCFAs. Wild-type, *LUC* RNAi, and *ACK* RNAi cell lines were grown in TYI-S-33 medium lacking glucose (designated as TYI basal medium) or TYI basal medium supplemented with 63 mM acetate, 24 mM propionate, or 23 mM butyrate (final concentration) for 72 h. These concentrations represent the estimated concentrations found in the colon based on total SCFA abundance and relative molar ratios. Growth of the *ACK* RNAi cell line was similar to that of the wild-type and the *LUC* RNAi cell line for all four media (Figure 6). Interestingly, all three cell lines showed slightly enhanced growth in the presence of added propionate versus TYI basal medium (*p*-value ≤ 0.01 for wild-type and *LUC* RNAi cell lines; *p*-value ≤ 0.001 for *ACK* RNAi cell line), but the presence of acetate or butyrate had no effect.

### 2.5. Oxidative and Nitrosative Stress Response Are Unaffected in the ACK RNAi Cell Line

**Figure 7.**
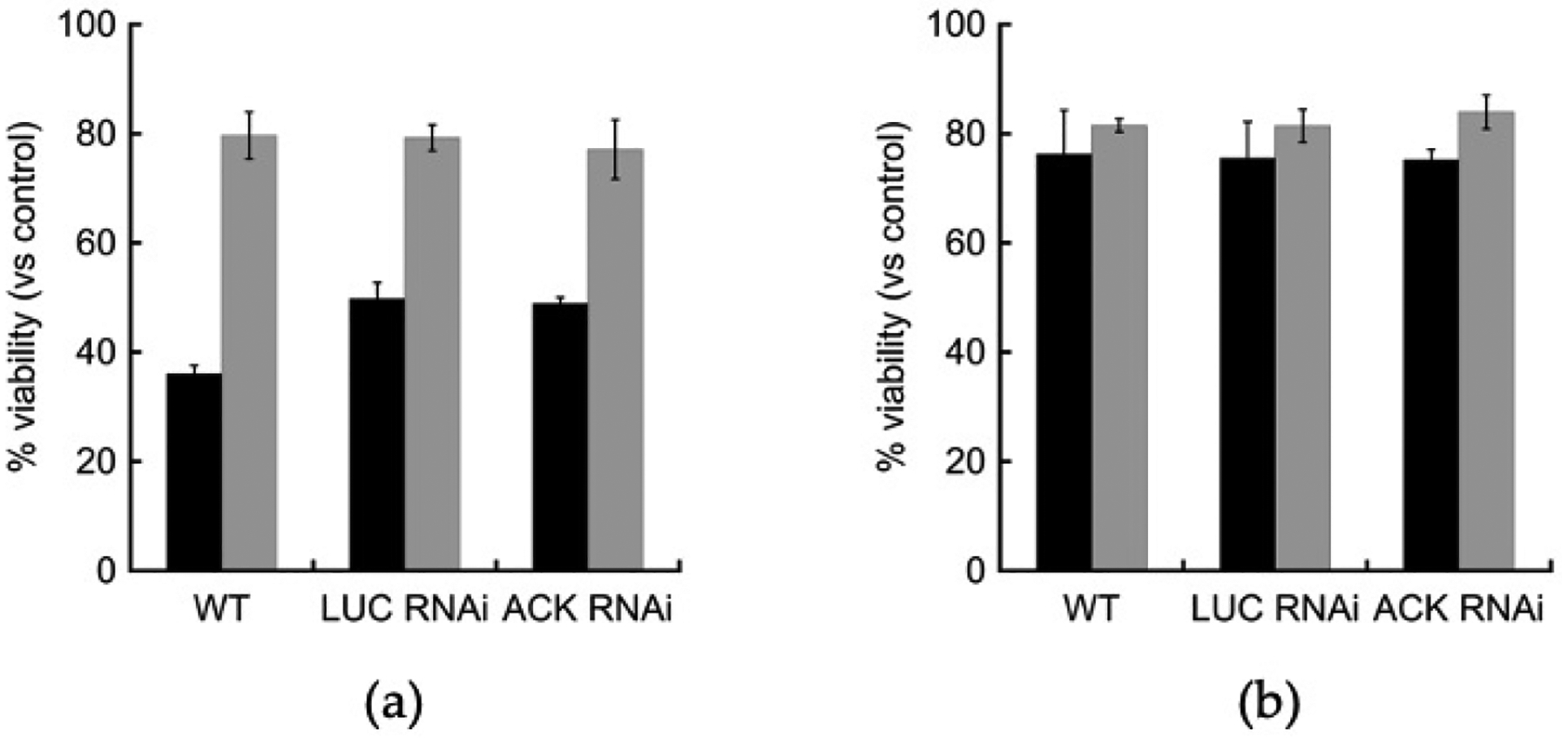
Effects of oxidative and nitrosative stress on *ACK* RNAi cells. Log-phase trophozoites in liquid medium were exposed to (**a**) 5 mM hydrogen peroxide (final concentration) for three hours at 37 °C to examine the effects of oxidative stress or to (**b**) 5 mM sodium nitroprusside (final concentration) for three hours at 37 °C to examine the effects of nitrosative stress. Viability for TYI glucose-grown (■) and TYI basal-grown (■) cells was determined using trypan blue exclusion. Values shown are the mean ± SD of three biological replicates. Unpaired *t*-test showed statistically significant difference in viability reduction in response to oxidative stress when grown on TYI glucose medium compared to growth on TYI basal medium.

We examined the effect of oxidative stress on an *ACK* RNAi cell line by exposing log-phase trophozoites to oxidative stress from exposure to hydrogen peroxide. Cells were grown in TYI glucose or TYI basal medium, exposed to 5 mM hydrogen peroxide (final concentration) for 3 h, and the change in viability was measured. The *ACK* RNAi cell line displayed similar changes in viability due to oxidative stress as the wild-type and *LUC* RNAi control cell lines (Figure 7a). When grown on TYI glucose medium, all three cell lines exhibited greater than 50% reduced viability after hydrogen peroxide exposure versus mock treated control cells. When grown on TYI basal medium though, the cell lines exhibited only ~20% reduced viability after hydrogen peroxide exposure versus mock treated control cells (Figure 7a).

To examine the effect of nitrosative stress on *ACK* RNAi cells, we exposed trophozoites grown in TYI glucose and TYI basal medium to 5 mM sodium nitroprusside (final concentration) for three hours at 37 °C to examine the effects of nitrosative stress on cell viability. The effect was similar for all three cell lines, which exhibited ~24% decreased viability versus mock treated cells when grown on TYI glucose medium and 16–18% reduced viability when grown in TYI basal medium (Figure 7b).

### 2.6. EhGAPDH Displays Acetyl Phosphate-Forming Activity in the Presence of ACK

Although ACK activity has been detected in *E. histolytica* cell extracts [16], the source of the acetyl phosphate substrate has remained unknown as the typical partner enzymes PTA and XFP are absent in *E. histolytica*. In 1954, Harting and Velick demonstrated that rabbit muscle and yeast glyceraldehyde 3-phosphate dehydrogenase (GAPDH) can catalyze the phosphorylation of acetaldehyde to form acetyl phosphate [24,25]. This enzyme is typically used to catalyze the phosphorylation of glyceraldehyde 3-phosphate to 1,3-bisphosphoglycerate in an NAD^+^-dependent reaction as part of glycolysis, and *E. histolytica* has three nearly identical genes encoding GAPDH.

We have purified recombinant EhGAPDH and demonstrated NADH-dependent activity with glyceraldehyde 3-phosphate (Jin Cho, personal communication). We also tested activity with acetaldehyde as a substrate (reaction 1), but no activity was detectable, despite varying the reaction conditions, substrate concentrations, and amount of enzyme (Jin Cho, personal communication). We next tested GAPDH activity with acetaldehyde in the presence of purified recombinant ACK as this coupling might draw the reaction in the direction of acetyl phosphate production. We used the purified recombinant enzymes EhACK (reaction 2a) [16,28] and the well-characterized *Methanosarcina thermophila* ACK (MtACK; reaction 2b) [29–32] for these coupled reactions. The coupled GAPDH-ACK reactions would be expected to proceed as follows:

(1) $$\text{GAPDH}\to \text{acetaldehyde}+{\text{P}}_{\text{i}}+\text{NADH}\to \text{acetyl phosphate}+{\text{NAD}}^{+}+{\text{H}}^{+}$$

(2a) $$\text{EhACK}\to \text{acetyl phosphate}+{\text{P}}_{\text{i}}\to \text{acetate}+{\text{PP}}_{\text{i}}$$

(2b) MtACK→acetyl phosphate+ADP→acetate+ATP

We measured GAPDH activity by following conversion of NADH to NAD^+^ with acetaldehyde as substrate. As shown in Table 1, GAPDH activity with acetaldehyde was observed only in the presence, but not absence, of ACK. To confirm that GAPDH’s use of acetaldehyde as a substrate is dependent on ACK enzymatic activity, we used both EhACK and MtACK as the coupling enzyme in the presence and absence of ADP (P_i_ is already present in the reaction as a substrate for GAPDH (Equation (1)). EhACK is P_i_-dependent (Equation (2a)) and the presence of ADP would not be expected to enhance GAPDH activity. As expected, the presence of ADP did not enhance GAPDH activity with EhACK as the coupling enzyme and instead inhibited activity, most likely because ADP acts as an inhibitor of EhACK [28]. MtACK’s activity is ADP-dependent (Equation (2b)) and we, thus, expected increased GAPDH activity in the presence of ADP. Indeed, GAPDH activity increased 50-fold in the presence versus absence of ADP when MtACK was used as the coupling enzyme. Thus, our results indicate that GAPDH activity with acetaldehyde is ACK-dependent and requires the appropriate phosphoryl donor for the ACK partner enzyme.

## 3. Discussion

EhACK has been hypothesized to provide supplemental pyrophosphate for the pyrophosphate-dependent glycolytic pathway in *E. histolytica*. In a comparison between the transcriptome of the virulent HM–1:IMSS and the nonvirulent Rahman cell lines, several glycolytic enzymes including PP_i_–PFK were found to be highly upregulated in HM–1:IMSS in both axenic culture and during contact with human colon explant [33]. This upregulation was thought to reflect the carbon metabolism needs during colonic mucosa degradation and tissue destruction during intestinal amoebiasis. EhACK is constitutively expressed in active trophozoites and at a slightly higher level in HM–1:IMSS versus the Rahman cell line [33–35]. These findings supported the possibility that EhACK could work in unison with PP_i_–PFK and PPDK to drive glycolysis, although the source of the acetyl phosphate substrate for ACK was unknown.

Here, we used RNAi gene silencing to investigate the role of ACK in *E. histolytica* metabolism. As acetate and ethanol are the two primary end products of glucose breakdown, we hypothesized that if ACK’s primary role is production PP_i_ for glycolysis, then growth on glucose would be affected in an *ACK* RNAi cell line. This was not the case though, as growth in low and high glucose media was similar for the *ACK* RNAi cell line and the control *LUC* RNAi and wild-type cell lines. Measurements of extracellular acetate and ethanol, the primary products of glucose metabolism in *E. histolytica* [11,12], revealed no difference between the three cell lines either. These results suggest that ACK is not a major contributor to acetate production, and thus not PP_i_ production either, at least not during standard growth on glucose as the main carbon and energy source. Pineda et al. similarly found that acetate and ethanol production were unaffected in an *ACK* RNAi cell line of the *E. histolytica* HM-1:IMSS G3 clone [36].

**Figure 8.**
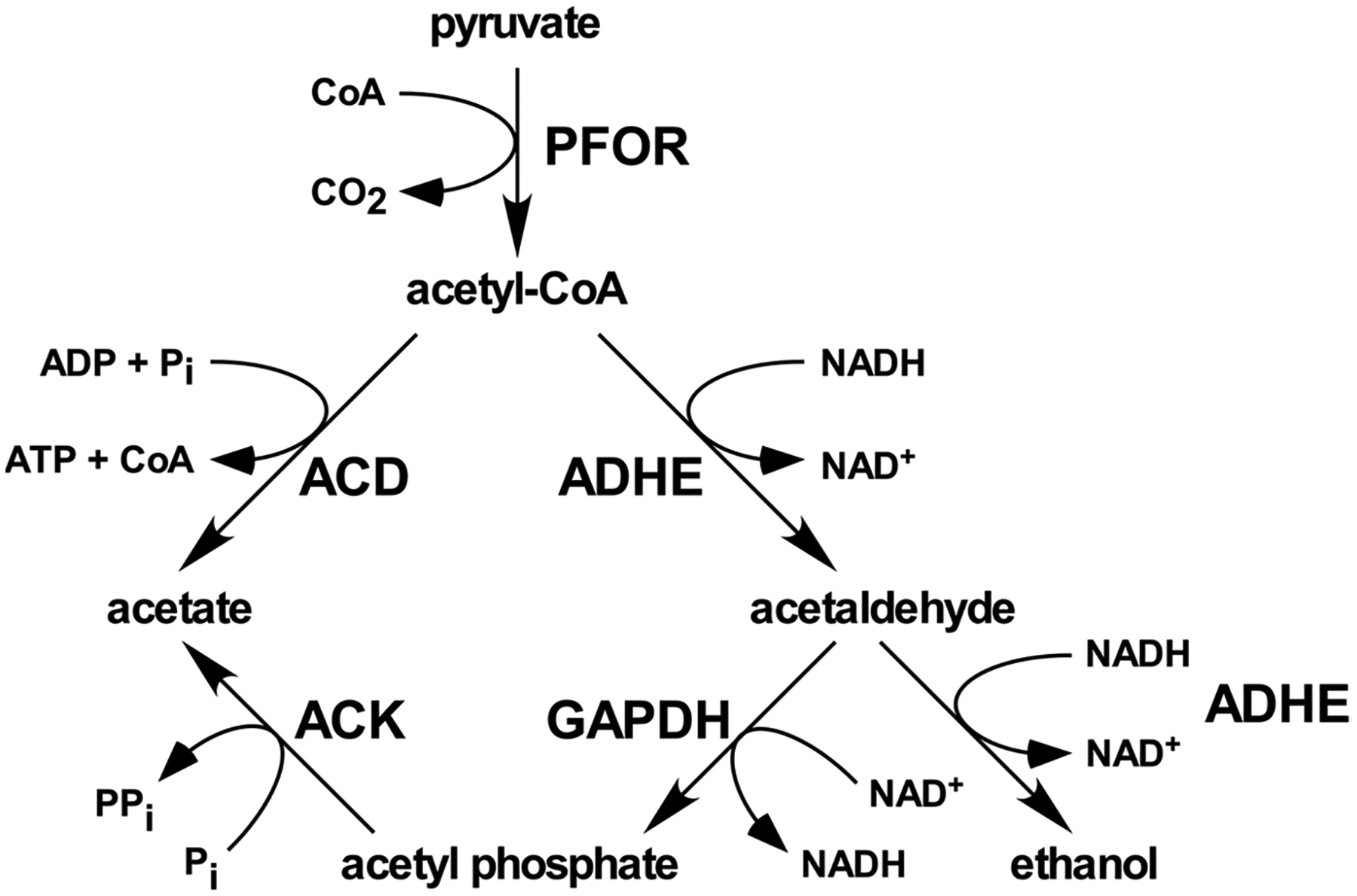
The extended glycolytic pathway in *E. histolytica* and the proposed role for ACK and GAPDH. Abbreviations are as follows: PFOR: pyruvate:ferredoxin oxidoreductase; ADHE: alcohol/aldehyde dehydrogenase; GAPDH: glyceraldehyde 3-phosphate dehydrogenase; ACK: acetate kinase; ACD: acetyl-CoA synthetase (ADP-forming).

Acetate and ethanol are produced as part of an extended glycolytic pathway in which pyruvate is first converted to acetyl-CoA, which is then broken down to acetate and ethanol by ADP-forming acetyl-CoA synthetase (ACD) and alcohol dehydrogenase (ADHE), respectively (Figure 8). Ethanol production by ADHE is a two-step reaction with acetaldehyde as an intermediate [10]. Each step requires one NADH for a total of two NADH per pyruvate converted to ethanol, yet glycolysis only generates one NADH molecule per pyruvate produced leading to an imbalance. Shunting pyruvate toward acetate production by ACD would help relieve this imbalance though as NADH is not required for this reaction. A 1:1 ratio of ethanol to acetate production would fully balance NADH production by glycolysis with NADH consumption by ADHE. However, we observed a ratio closer to 2:1 ethanol:acetate, similar to previously published values [11], suggesting an imbalance would be present.

We propose that EhACK plays a role in the extended glycolytic pathway in maintaining the proper NAD^+^/NADH ratio during growth on glucose. In this proposed pathway, shown in Figure 8, when NADH levels are insufficient to continue ethanol production, acetaldehyde produced in the first step of the ADHE reaction would be released to GAPDH, which would then convert it to acetyl phosphate for use by ACK. NADH would be generated in this process to restore balance and allow ethanol production to continue. This would be expected to be an overflow pathway that would not be a major contributor to acetate production but would help maintain proper balance between the acetate and ethanol production.

Consistent with this proposed pathway, we showed that GAPDH can indeed use acetaldehyde as a substrate, but only when ACK was present to draw the reaction toward acetyl phosphate production. We observed that an *ACK* RNAi cell line has increased intracellular acetyl-CoA, suggesting that the inability to shunt acetaldehyde into a GAPDH-ACK pathway causes a slowdown of the ADHE pathway. Pineda et al. were unable to detect intracellular acetyl phosphate in *E. histolytica* grown in TYI glucose medium [36], but this is consistent with the proposed pathway in which the acetyl phosphate would be quickly converted to acetate by ACK.

We induced oxidative and nitrosative stress in *E. histolytica* trophozoites to observe the effect that reduced ACK activity would have on cell viability under variable glucose conditions. We observed that induction of oxidative stress had a more negative effect on viability in the presence of glucose versus the absence of glucose for all three cell lines, suggesting that *E. histolytica* tolerates oxidative stress better when glycolysis is less active. Reduction in ACK activity in the *ACK* RNAi cell line did not confer an advantage or disadvantage for oxidative stress tolerance. Given that ACK does not seem to be a major contributor to PP_i_ production and cell growth, this result was not unexpected. Our data are consistent with previous research that showed ACK was not significantly differentially expressed in cells exposed to oxidative stress [37]. Likewise, reduction in ACK activity did not confer an advantage or disadvantage for nitrosative stress tolerance either; however, *ACK* RNAi cells tolerated nitrosative stress better in the absence of glucose than in glucose-containing medium. The reason for this is not known.

Finally, we examined whether acetate, propionate, or butyrate affected growth of *E. histolytica* in the absence of glucose. Growth increased when propionate, but not acetate or butyrate, was present. That *ACK* gene silencing did not influence increased growth with propionate is not surprising, as the EhACK reaction proceeds strongly in the direction of acetate/propionate production rather than utilization [16,17].

## 4. Materials and Methods

### 4.1. Chemicals and Reagents

Chemicals were purchased from Qiagen (Valencia, CA, USA) Promega (Madison, WI, USA), Sigma-Aldrich (St. Louis, MO, USA), VWR International (Radnor, PA, USA), Gold Biotechnology (Olivette, MO, USA), Fisher Scientific (Waltham, MA, USA), EMD Millipore (Burlington, MA, USA), and Life Technologies (Carlsbad, CA, USA). Penicillin-streptomycin solution and Diamond vitamins were purchased from Life Technologies (Carlsbad, CA, USA) and heat-inactivated adult bovine serum from GeminiBio (Sacramento, CA, USA). Restriction enzymes were purchased from New England Biolabs (Ipswich, MA, USA). Primers were purchased from Integrated DNA Technologies (Coralville, IA, USA).

### 4.2. Cell Lines and Culture Conditions

*E. histolytica* HM-1:IMSS was grown axenically at 37 °C in Diamond’s TYI-S-33 medium [38] (17.95 g tryptone, 9.66 g yeast extract, 9.2 g glucose, 1.84 g NaCl, 0.92 g K_2_HPO_4_, 1.15 g cysteine, 0.178 g ascorbic acid, 0.0194 g ammonium ferric chloride, 15% *v*/*v* adult bovine serum, 1.73% *v*/*v* penicillin-streptomycin, and 2.62% *v*/*v* Diamond vitamins per liter, final pH 6.8). Trophozoites in log phase growth were used for all experiments. Modifications to media formulations and their designations are as follows: TYI glucose, standard TYI-S-33 medium (which contains 50 mM added glucose as shown above); TYI basal, TYI-S-33 medium without added glucose; and TYI low glucose, TYI-S-33 medium with the added glucose reduced to 10 mM. Growth on acetate, propionate, or butyrate was performed in TYI basal medium supplemented with 63 mM acetate (TYI acetate), 24 mM propionate (TYI propionate), or 23 mM butyrate (TYI butyrate). These concentrations of short chain fatty acids represent those found in the large intestine [27].

Growth curves were determined by counting cells every 24 h using a Luna Automated counter (Logos Biosystem, Annandale, VA, USA). Trypan blue exclusion was used to distinguish viable from dead cells [39]. Growth curves were performed with three biological replicates and values for each time point represent the mean ± standard deviation.

### 4.3. Construct Cloning and Transfection

The plasmid pKT3M (kindly provided by Dr. Upinder Singh, Stanford University, Stanford, CA, USA) [26] was used for construction of an *ACK* RNAi cell line. The full-length *Entamoeba histolytica ACK* coding sequence (EHI_170010) was PCR-amplified from *E. histolytica* genomic DNA using KOD Hot Start Polymerase (EMD Millipore, Billerica, MA, USA). The PCR product was cloned into *Avr*II and *Xho*I restriction sites to replace the resident luciferase (*LUC*) control gene. The final construct was confirmed by sequencing.

*E. histolytica* trophozoites were transfected with the *ACK* RNAi construct or the control pKT3M plasmid by electroporation as described previously [40,41]. A total of 2.4 × 10^6^ cells were electroporated with 100 μg of DNA using two consecutive pulses at 1.2 kV and 25 uF and inoculated into TYI-S-33 medium. Transfectants were selected after two days by adding G418 to the medium to a final concentration of 6 μg/mL. Stable transfectants were maintained under this level of G418 selection. Primers used for construction of the *ACK* RNAi plasmid are listed Table 2.

### 4.4. Reverse Transcriptase PCR (RT-PCR)

RT-PCR primers are listed in Table 2. RNA was isolated from 2 × 10^6^ trophozoites using the RNeasy mini kit (Qiagen, Valencia, CA, USA) or Trizol reagent according to the manufacturers’ instructions. RT-PCR was employed to determine whether the *EhACK* gene was silenced in the *ACK* RNAi cell line. RT-PCR was performed using the One-Step RT-PCR kit (Qiagen) using 30 cycles. RNA levels were normalized for comparison using the small subunit ribosomal RNA gene (accession number: X61116) as previously described [42].

### 4.5. Enzyme Assays

*EhACK* knockdown was confirmed by measuring ACK activity in cell lysates. A total of 4 × 10^6^ cells were harvested by centrifugation and washed twice in phosphate buffered saline (PBS). Cells were resuspended in 25 mM Tris, 150 mM NaCl (pH 7.4) and lysed by vortexing with acid-washed beads for 1 min, followed by 1 min on ice. This cycle was repeated three times. The lysates were centrifuged at 5000× *g* for 15 min and the supernatant was retained.

ACK activity was measured using the reverse hydroxamate assay as previously described [16]. Enzyme activity was assessed in a 300 μL reaction containing 50 mM sodium phosphate, 2 mM acetyl phosphate, 100 mM Tris-HCl (pH 7.0), 10 mM MgCl_2_, and 50 μL cell lysate at 37 °C. Reactions were terminated after 30 min by adding 100 μL of the development solution (0.92 M trichloroacetic acid, 250 mM FeCl_3_, and 2.5 N HCl). Absorbance at 540 nM was measured using a Synergy Epoch microplate reader (Biotek, Winooski, VT, USA).

GAPDH activity with acetaldehyde as substrate was determined using a spectrophotometric assay that measures the conversion of NAD^+^ to NADH. The reaction mix contained 25 mM Tris-HCl (pH 7.0), 2.5 mM potassium phosphate buffer (pH 7.0), 1 mM NAD^+^, and 1 mM DTT. Reactions were performed in the presence or absence of recombinant *E. histolytica* ACK (EhACK) [16] or *Methanosarcina thermophila* ACK (MtACK) [29,30] as a coupling enzyme to convert acetyl phosphate to acetate to draw the reaction forward. The reaction was initiated by addition of acetaldehyde to a final concentration of 500 mM and the absorbance change at 340 nm was measured to monitor the conversion of NAD^+^ to NADH at 37 °C using a Synergy Epoch microplate reader (Biotek, Winooski, VT, USA).

Total protein concentration in cell lysates was measured using the Bradford assay [43,44] with bovine serum albumin as the standard.

### 4.6. Intracellular Metabolite Analysis

*E. histolytica* intracellular metabolites were extracted using an adapted methanol extraction method [45] from 1 × 10^6^ log phase trophozoites. Cells were harvested by centrifugation, washed three times in ice cold 5% mannitol solution, and resuspended in 1.5 mL ice cold 100% methanol. Trophozoites were lysed using three freeze–thaw cycles in which cells were frozen in liquid nitrogen for 5 min and thawed on dry ice for 10 min. The lysate was centrifuged at 10,000× *g* for 5 min and the supernatant isolated. Samples were analyzed at the Clemson Multi-User Analytic Lab via LC-MS/MS. Intracellular metabolite concentrations were calculated by assuming *E. histolytica* trophozoites have an intracellular volume of 1.3 μL per 1 × 10^6^ cells as previously reported [45].

### 4.7. Extracellular Metabolite Analysis

Acetate in spent TYI glucose medium from 48 h cultures was measured using the hydroxamate assay in which acetate is converted to acetyl phosphate, which is then converted to an acetyl hydroxamate complex that can be detected spectrophotometrically [46]. Reactions (300 μL) contained 150 μL reaction mix [100 mM Tris-HCL (pH 7.0), 600 mM hydroxylamine HCl (pH 7.0), 10 mM MgCl_2_, 10 mM ATP, 100 μg recombinant MtACK], and 150 μL spent medium. Reactions were incubated at 37 °C and terminated after 30 min by adding 600 μL of development solution (0.92 M trichloroacetic acid, 250 mM FeCl_3_, and 2 N HCl). Samples were centrifuged at 10,000× *g* for 5 min to pellet precipitated proteins. The absorbance at 540 nM was measured using a Synergy Epoch microplate reader (Biotek, Winooski, VT, USA) and compared against a standard curve generated with acetyl phosphate.

Ethanol in spent TYI glucose medium from 48-h cultures was measured using the EnzyChrom^™^ Ethanol Assay Kit (BioAssay Systems, Hayward, CA, USA), which is based on the alcohol dehydrogenase-catalyzed oxidation of ethanol. Reactions were prepared according to the manufacturer’s instructions and terminated after 30 min. Absorbance at 565 nM was measured using a Synergy Epoch microplate reader (Biotek, Winooski, VT, USA).

### 4.8. Oxidative Stress and Nitrosative Stress Induction

The effect of oxidative or nitrosative stress on *E. histolytica* trophozoites was determined by exposing trophozoites to hydrogen peroxide or sodium nitroprusside in liquid culture and then assessing change in cell viability. Log-phase trophozoites were harvested and counted using trypan blue exclusion [39] on a Luna Automated Counter (Logos Biosystem, Annandale, VA, USA). Samples containing 3.5 × 10^4^ live cells resuspended in fresh, prewarmed TYI glucose medium were prepared for each culture. For oxidative stress, hydrogen peroxide was added to final concentration of 5 mM to one sample and an equal volume of water was added to the control sample. For nitrosative stress, sodium nitroprusside was added to a final concentration of 5 mM to one sample and an equal volume of water was added to the control sample. Sample tubes were rotated for 3 h at 37 °C, and cell viability was determine using trypan blue exclusion. The difference in cell viability between each sample and its control was recorded to determine the effect of oxidative or nitrosative stress on each cell type. Three biological replicates were performed for each cell type.

## 5. Conclusions

Contrary to our starting hypothesis, we found that ACK is unlikely to be a major contributor to acetate production or the PP_i_ pool in *E. histolytica*. Instead, our results suggest that ACK plays a role in the extended glycolytic pathway in *E. histolytica* to provide a mechanism for maintaining proper NAD^+^/NADH balance during glycolysis and ethanol production and, as such, could potentially play a role in partitioning of acetyl-CoA between acetate and ethanol production. Such a function may be essential under some conditions but not others, but this remains to be investigated.

## Figures and Tables

**Table 1.** GAPDH activity with acetaldehyde in the presence and absence of ACK.

	Specific Activity (nmol min^−1^ mg^−1^)^1^	
Enzymes	No ADP	10 mM ADP
GAPDH	ND	ND
GAPDH + EhACK	14.1 ± 0.60	7.04 ± 0.90
GAPDH + MtACK	2.29 ± 0.16	128 ± 2.33

1 All assays were performed in the presence of 2.5 mM P_i_. ND—not detected.

**Table 2.** Primers for RNAi construct generation and RT-PCR confirmation.

Cloning Primers	
*EhACK* RNAi F	5′ CTACCTAGGATGTCTAACGTACTAATATTCAACG
*EhACK* RNAi R	5′ CTACTCGAGTTAAAACTGAAATAATTCTTTTCCTTTTTGTAA
RT-PCR primers	
*EhACK* RTPCRF	5′ AGGGTAAATGTTACAGGAACAGA
*EhACK* RTPCR R	5′ TGGTGCCACACAAACTTGAAC
ssrRNA F	5′-AGGCGCGTAAATTACCCACTTTCG
ssRNA R	5′-CACCAGACTTGCCCTCCAATTGAT

## Data Availability

Data presented in this study are available within the article.

## References

[1] World Health Organization; Foodborne Disease Burden Epidemiology Reference Group. WHO Estimates of the Global Burden of Foodborne Diseases; World Health Organization: Geneva, Switzerland, 2015; p. 11, 254p.

[2] Baxt LA; Singh U New insights into *Entamoeba histolytica* pathogenesis. Curr. Opin. Infect. Dis 2008, 21, 489–494. 18725798 10.1097/QCO.0b013e32830ce75f PMC2688559

[3] Haque R; Huston CD; Hughes M; Houpt E; Petri WA Jr. Amebiasis. N. Engl. J. Med 2003, 348, 1565–1573. 12700377 10.1056/NEJMra022710

[4] Stanley SL Jr. Amoebiasis. Lancet 2003, 361, 1025–1034. 12660071 10.1016/S0140-6736(03)12830-9

[5] Anderson IJ; Loftus BJ *Entamoeba histolytica*: Observations on metabolism based on the genome sequence. Exp. Parasitol 2005, 110, 173–177. 15955308 10.1016/j.exppara.2005.03.010

[6] Clark CG; Alsmark UC; Tazreiter M; Saito-Nakano Y; Ali V; Marion S; Weber C; Mukherjee C; Bruchhaus I; Tannich E; Structure and content of the *Entamoeba histolytica* genome. Adv. Parasitol 2007, 65, 51–190. 18063096 10.1016/S0065-308X(07)65002-7

[7] Reeves RE; Serrano R; South DJ 6-phosphofructokinase (pyrophosphate). Properties of the enzyme from *Entamoeba histolytica* and its reaction mechanism. J. Biol. Chem 1976, 251, 2958–2962. 178659

[8] Saavedra E; Encalada R; Pineda E; Jasso-Chavez R; Moreno-Sanchez R Glycolysis in *Entamoeba histolytica*. Biochemical characterization of recombinant glycolytic enzymes and flux control analysis. FEBS J. 2005, 272, 1767–1783. 15794763 10.1111/j.1742-4658.2005.04610.x

[9] Saavedra-Lira E; Perez-Montfort R Energy production in *Entamoeba histolytica*: New perspectives in rational drug design. Arch. Med. Res 1996, 27, 257–264. 8854380

[10] Lo HS; Reeves RE Pyruvate-to-ethanol pathway in *Entamoeba histolytica*. Biochem. J 1978, 171, 225–230. 25658 10.1042/bj1710225 PMC1184151

[11] Montalvo FE; Reeves RE; Warren LG Aerobic and anaerobic metabolism in *Entamoeba histolytica*. Exp. Parasitol 1971, 30, 249–256. 4331796 10.1016/0014-4894(71)90089-0

[12] Reeves RE; Warren LG; Susskind B; Lo HS An energy-conserving pyruvate-to-acetate pathway in *Entamoeba histolytica*. Pyruvate synthase and a new acetate thiokinase. J. Biol. Chem 1977, 252, 726–731. 13076

[13] Pineda E; Encalada R; Olivos-Garcia A; Nequiz M; Moreno-Sanchez R; Saavedra E The bifunctional aldehyde-alcohol dehydrogenase controls ethanol and acetate production in *Entamoeba histolytica* under aerobic conditions. FEBS Lett. 2013, 587, 178–184. 23201265 10.1016/j.febslet.2012.11.020

[14] Pineda E; Encalada R; Rodriguez-Zavala JS; Olivos-Garcia A; Moreno-Sanchez R; Saavedra E Pyruvate:ferredoxin oxidoreductase and bifunctional aldehyde-alcohol dehydrogenase are essential for energy metabolism under oxidative stress in *Entamoeba histolytica*. FEBS J. 2010, 277, 3382–3395. 20629749 10.1111/j.1742-4658.2010.07743.x

[15] Espinosa A; Yan L; Zhang Z; Foster L; Clark D; Li E; Stanley SL Jr. The bifunctional *Entamoeba histolytica* alcohol dehydrogenase 2 (EhADH2) protein is necessary for amebic growth and survival and requires an intact C-terminal domain for both alcohol dahydrogenase and acetaldehyde dehydrogenase activity. J. Biol. Chem 2001, 276, 20136–20143. 11274185 10.1074/jbc.M101349200 PMC4816598

[16] Fowler ML; Ingram-Smith C; Smith KS Novel pyrophosphate-forming acetate kinase from the protist *Entamoeba histolytica*. Eukaryot. Cell 2012, 11, 1249–1256. 22903977 10.1128/EC.00169-12 PMC3485911

[17] Reeves RE; Guthrie JD Acetate kinase (pyrophosphate). A fourth pyrophosphate-dependent kinase from *Entamoeba histolytica*. Biochem. Biophys. Res. Commun 1975, 66, 1389–1395. 172079 10.1016/0006-291x(75)90513-6

[18] Jones CP; Ingram-Smith C Biochemical and kinetic characterization of the recombinant ADP-forming acetyl coenzyme A synthetase from the amitochondriate protozoan *Entamoeba histolytica*. Eukaryot. Cell 2014, 13, 1530–1537. 25303954 10.1128/EC.00192-14 PMC4248687

[19] Wolfe AJ The acetate switch. Microbiol. Mol. Biol. Rev 2005, 69, 12–50. 15755952 10.1128/MMBR.69.1.12-50.2005 PMC1082793

[20] Terlesky KC; Barber MJ; Aceti DJ; Ferry JG EPR properties of the Ni-Fe-C center in an enzyme complex with carbon monoxide dehydrogenase activity from acetate-grown *Methanosarcina thermophila*. Evidence that acetyl-CoA is a physiological substrate. J. Biol. Chem 1987, 262, 15392–15395. 2824458

[21] Singh-Wissmann K; Ferry JG Transcriptional regulation of the phosphotransacetylase-encoding and acetate kinase-encoding genes (pta and ack) from *Methanosarcina thermophila*. J. Bacteriol 1995, 177, 1699–1702. 7896690 10.1128/jb.177.7.1699-1702.1995 PMC176795

[22] Ingram-Smith C; Martin SR; Smith KS Acetate kinase: Not just a bacterial enzyme. Trends Microbiol. 2006, 14, 249–253. 16678422 10.1016/j.tim.2006.04.001

[23] Yang W; Catalanotti C; D’Adamo S; Wittkopp TM; Ingram-Smith CJ; Mackinder L; Miller TE; Heuberger AL; Peers G; Smith KS; Alternative acetate production pathways in *Chlamydomonas reinhardtii* during dark anoxia and the dominant role of chloroplasts in fermentative acetate production. Plant Cell 2014, 26, 4499–4518. 25381350 10.1105/tpc.114.129965 PMC4277214

[24] Harting J; Velick SF Transfer reactions of acetyl phosphate catalyzed by glyceraldehyde-3-phosphate dehydrogenase. J. Biol. Chem 1954, 207, 867–878. 13163072

[25] Harting J; Velick SF Acetyl phosphate formation catalyzed by glyceraldehyde-3-phosphate dehydrogenase. J. Biol. Chem 1954, 207, 857–865. 13163071

[26] Morf L; Pearson RJ; Wang AS; Singh U Robust gene silencing mediated by antisense small RNAs in the pathogenic protist *Entamoeba histolytica*. Nucleic Acids Res. 2013, 41, 9424–9437. 23935116 10.1093/nar/gkt717 PMC3814356

[27] Cummings JH; Pomare EW; Branch WJ; Naylor CP; Macfarlane GT Short chain fatty acids in human large intestine, portal, hepatic and venous blood. Gut 1987, 28, 1221–1227. 3678950 10.1136/gut.28.10.1221 PMC1433442

[28] Dang T; Ingram-Smith C Investigation of pyrophosphate versus ATP substrate selection in the *Entamoeba histolytica* acetate kinase. Sci. Rep 2017, 7, 5912. 28724909 10.1038/s41598-017-06156-5 PMC5517563

[29] Aceti DJ; Ferry JG Purification and characterization of acetate kinase from acetate-grown *Methanosarcina thermophila*. Evidence for regulation of synthesis. J. Biol. Chem 1988, 263, 15444–15448. 2844814

[30] Latimer MT; Ferry JG Cloning, sequence analysis, and hyperexpression of the genes encoding phosphotransacetylase and acetate kinase from *Methanosarcina thermophila*. J. Bacteriol 1993, 175, 6822–6829. 8226623 10.1128/jb.175.21.6822-6829.1993 PMC206805

[31] Ingram-Smith C; Gorrell A; Lawrence SH; Iyer P; Smith K; Ferry JG Characterization of the acetate binding pocket in the *Methanosarcina thermophila* acetate kinase. J. Bacteriol 2005, 187, 2386–2394. 15774882 10.1128/JB.187.7.2386-2394.2005 PMC1065240

[32] Ingram-Smith C; Barber RD; Ferry JG The role of histidines in the acetate kinase from *Methanosarcina thermophila*. J. Biol. Chem 2000, 275, 33765–33770. 10958794 10.1074/jbc.M005303200

[33] Thibeaux R; Weber C; Hon CC; Dillies MA; Ave P; Coppee JY; Labruyere E; Guillen N Identification of the virulence landscape essential for *Entamoeba histolytica* invasion of the human colon. PLoS Pathog. 2013, 9, e1003824. 24385905 10.1371/journal.ppat.1003824 PMC3868522

[34] Ehrenkaufer GM; Haque R; Hackney JA; Eichinger DJ; Singh U Identification of developmentally regulated genes in *Entamoeba histolytica*: Insights into mechanisms of stage conversion in a protozoan parasite. Cell Microbiol. 2007, 9, 1426–1444. 17250591 10.1111/j.1462-5822.2006.00882.x

[35] Hon CC; Weber C; Sismeiro O; Proux C; Koutero M; Deloger M; Das S; Agrahari M; Dillies MA; Jagla B; Quantification of stochastic noise of splicing and polyadenylation in *Entamoeba histolytica*. Nucleic Acids Res. 2013, 41, 1936–1952. 23258700 10.1093/nar/gks1271 PMC3561952

[36] Pineda E; Vazquez C; Encalada R; Nozaki T; Sato E; Hanadate Y; Nequiz M; Olivos-Garcia A; Moreno-Sanchez R; Saavedra E Roles of acetyl-CoA synthetase (ADP-forming) and acetate kinase (PP_i_-forming) in ATP and PP_i_ supply in *Entamoeba histolytica*. Biochim. Biophys. Acta 2016, 1860, 1163–1172. 26922831 10.1016/j.bbagen.2016.02.010

[37] Vicente JB; Ehrenkaufer GM; Saraiva LM; Teixeira M; Singh U *Entamoeba histolytica* modulates a complex repertoire of novel genes in response to oxidative and nitrosative stresses: Implications for amebic pathogenesis. Cell Microbiol. 2009, 11, 51–56. 18778413 10.1111/j.1462-5822.2008.01236.x PMC3418052

[38] Diamond LS; Harlow DR; Cunnick CC A new medium for the axenic cultivation of *Entamoeba histolytica* and other *Entamoeba*. Trans. R. Soc. Trop. Med. Hyg 1978, 72, 431–432. 212851 10.1016/0035-9203(78)90144-x

[39] Strober W Trypan blue exclusion test of cell viability. Curr. Protoc. Immunol 2001, 21, A.3B.1–A.3B.2. 10.1002/0471142735.ima03bs21 18432654

[40] Hamann L; Nickel R; Tannich E Transfection and continuous expression of heterologous genes in the protozoan parasite *Entamoeba histolytica*. Proc. Natl. Acad. Sci. USA 1995, 92, 8975–8979. 7568055 10.1073/pnas.92.19.8975 PMC41090

[41] Vines RR; Purdy JE; Ragland BD; Samuelson J; Mann BJ; Petri WA Jr. Stable episomal transfection of *Entamoeba histolytica*. Mol. Biochem. Parasitol 1995, 71, 265–267. 7477110 10.1016/0166-6851(95)00057-8

[42] Koushik AB; Welter BH; Rock ML; Temesvari LA A genomewide overexpression screen identifies genes involved in the phosphatidylinositol 3-kinase pathway in the human protozoan parasite *Entamoeba histolytica*. Eukaryot. Cell 2014, 13, 401–411. 24442890 10.1128/EC.00329-13 PMC3957588

[43] Bradford MM A rapid and sensitive method for the quantitation of microgram quantities of protein utilizing the principle of protein-dye binding. Anal. Biochem 1976, 72, 248–254. 942051 10.1016/0003-2697(76)90527-3

[44] Stoscheck CM Quantitation of protein. Methods Enzymol. 1990, 182, 50–68. 2314256 10.1016/0076-6879(90)82008-p

[45] Neubauer S; Chu DB; Marx H; Sauer M; Hann S; Koellensperger G LC-MS/MS-based analysis of coenzyme A and short-chain acyl-coenzyme A thioesters. Anal. Bioanal. Chem 2015, 407, 6681–6688. 26168961 10.1007/s00216-015-8825-9

[46] Rose IA; Grunberg-Manago M; Korey SR; Ochoa S Enzymatic phosphorylation of acetate. J. Biol. Chem 1954, 211, 737–756. 13221579

